# Contemporary Chronic Limb-Threatening Ischemia Care in the United States—Part 2: Designing Clinical Device Trials

**DOI:** 10.1016/j.jscai.2025.103934

**Published:** 2025-11-03

**Authors:** Eric A. Secemsky, Ehrin J. Armstrong, Venita Chandra, Raghu Kolluri, Saher S. Sabri, Niten Singh

**Affiliations:** a Division of Cardiology, Beth Israel Deaconess Medical Center, Boston, Massachusetts; b Department of Interventional Cardiology, Advanced Heart and Vein Center, Denver, Colorado; c Division of Vascular Surgery, Stanford University Medical Center, Stanford, California; d Cardiovascular Service Line – OhioHealth Riverside Methodist Hospital, Columbus, Ohio; e Division of Interventional Radiology, MedStar Georgetown University Hospital, Washington, DC; f Division of Vascular Surgery, Department of Surgery, Harborview Medical Center, Seattle, Washington

**Keywords:** chronic limb-threatening ischemia, clinical trials, end points, randomized registry controlled trials, registry studies, target trial emulation

## Abstract

Head-to-head research comparing invasive revascularization strategies for chronic limb-threatening ischemia (CLTI) is sparse, partly due to challenges in conducting randomized controlled trials in the CLTI space. These include the expense of head-to-head trials, optimizing patient selection criteria for real-world applicability, and identifying optimal study end points. The Vascular InterVentional Advances (VIVA) Foundation, a 501(c)(3) not-for-profit organization, convened a Vascular Leaders Forum to initiate an open, multispecialty collaborative discussion of these challenges and ways to optimize the design of medical device trials in CLTI. This article summarizes the current landscape of clinical studies of CLTI revascularization strategies and options for designing comparative trials proposed by representatives from vascular surgery, interventional cardiology, interventional radiology, vascular medicine, podiatry, the U.S. Food and Drug Administration, and a medical device manufacturer. Four broad areas to optimize comparative trials of CLTI interventions were identified. First, primary end points should be carefully chosen with attention to clinical, patient-centric, imaging, and hierarchical considerations; standardization; and inclusion of guideline-directed medical therapy. Second, broader eligibility criteria can expand and hasten enrollment and are important for gathering evidence on outcomes in medically complex patients often encountered in real-world practice. Third, extending the primary end point timing to 12 months with additional follow-up out to 24 to 60 months would accommodate a longer period of device evaluation and the ability to enrich clinical end-point rates. Finally, innovative pragmatic trial designs and statistical methodologies are needed to conduct comprehensive, cost effective, relevant trials with sufficient statistical power and without prohibitively large sample sizes and study durations.

## Introduction

Chronic limb-threatening ischemia (CLTI) affects approximately 11% of patients with peripheral artery disease (PAD) and represents the most severe end of the PAD spectrum.^1^^,^^2^ CLTI is defined as PAD in combination with rest pain, lower limb ulceration, or gangrene of more than 2 weeks duration.^1^ It is associated with significant mortality, limb loss, pain, health care costs, and diminished quality of life (QOL).^1^, ^2^, ^3^, ^4^, ^5^ With the worldwide prevalence of PAD increasing over the last 3 decades,^6^, ^7^, ^8^ determining the most efficacious revascularization approaches for CLTI is imperative. The combination of endovascular or surgical revascularization along with best medical therapy, wound care, smoking cessation, diet, and exercise aims to improve limb salvage and survival and maximize QOL.^1^^,^^9^ However, there is a dearth of head-to-head research comparing CLTI revascularization strategies and combinations of approaches. Barriers to conducting randomized controlled trials (RCTs) in the CLTI space are substantial. These include the expense of head-to-head trials, under-optimized patient selection criteria for real-world applicability, and difficulty identifying the appropriate study end points.

The Vascular InterVentional Advances (VIVA) Foundation convened a Vascular Leaders Forum on April 12, 2024, in Washington, DC, to initiate an open, multispecialty collaborative discussion of these challenges and ways to optimize the design of CLTI revascularization trials. The attendees comprised 52 US stakeholders, including representatives of vascular surgery (29%), interventional cardiology (26%), interventional radiology (24%), vascular medicine (7%), and podiatry (2%), as well as 2 U.S. Food and Drug Administration (FDA) representatives, 1 representative from a large device manufacturer, 1 patient advocate, and 1 CLTI patient and their caregiver.

This article explores the current landscape of clinical studies of revascularization for CLTI and options for designing comparative device trials in this space.

## Device approval studies: What remains missing?

Unique to peripheral vascular intervention is the number of devices that fall into the FDA 510(k) regulatory approval pathway. This approval pathway evaluates investigational medical devices by comparing their characteristics to a predicate (ie, already approved) device. The 510(k) pathway lacks requirements for head-to-head comparison of an investigational device or technology with an approved device or technology, evaluation of long-term outcomes, annual reporting, and prior approval for manufacturing changes. The goal of the 510(k) pathway is to expedite innovation and time to market for lower-risk devices, focusing on safety over efficacy. Among the currently approved devices for PAD treatment, plain balloon angioplasty, thrombectomy, and atherectomy are all examples of devices approved through the 510(k) pathway. A key challenge with this regulatory pathway is the dearth of comparative evidence once these devices come to market. This is particularly the case for atherectomy, which has achieved notable growth in use in the US without the support of high-quality evidence.^10^^,^^11^ Furthermore, with the 510(k) pathway, there are no additional postmarket data collection requirements unless a safety issue is identified, and no requirement for, or regulation of, on-label studies for reimbursement and quality metrics.

Although most advocate for investment in head-to-head device trials, because this is not part of the regulatory approval pathway for many devices, the lack of a mandate to conduct these studies and the resultant costs to do so have hampered evidence generation. Without a regulatory incentive, device manufacturers are unlikely to make the substantial investment necessary to generate such randomized trial data. Obtaining funding from public organizations such as the National Institutes of Health can be cumbersome and time-consuming. A commitment to generating RCT evidence in the postmarket setting is essential, but how to best accomplish this remains unclear.

## Postapproval device trials: What are the possibilities?

Most evidence strategies for approved devices have focused on prospective registries rather than RCT. These studies collect real-world evidence to support indication expansion and clinical decision-making, advance the quality of clinical evidence, evaluate treatments in underrepresented populations (eg, the ELEGANCE registry^12^), collect important patient-reported data such as improved mobility and reduced pain or discomfort (eg, the SUCCESS PTA study^13^), and test innovative ways to analyze big data (eg, TRUVETA deidentified electronic health records). In general, registry studies can be larger and cheaper and can provide real-world evidence in unselected PAD populations that includes important patient-reported outcomes such as improved mobility and reduced pain or discomfort, as reported in the SUCCESS PTA study. Generally, the FDA does not request postapproval studies for medical devices without a specific reason, such as for some premarket approval applications or to address safety concerns.^14^, ^15^, ^16^ At the Vascular Leaders Forum, FDA representatives shared some preferred characteristics of postapproval trials in CLTI (Table 1).

**Table 1:** FDA’s preferred characteristics for postapproval CLTI clinical trials.

Characteristics
• Randomized controlled trial is optimal • Well designed and operated • Diverse enrollment • Prespecified but flexible statistical analysis plan • Consistent definitions • Objective assessments • Wound care assessments • Standardized treatment that mirrors clinical practice

CLTI, chronic limb-threatening ischemia; FDA, U.S. Food and Drug Administration; RCT, randomized controlled trial.

**Figure 1:**
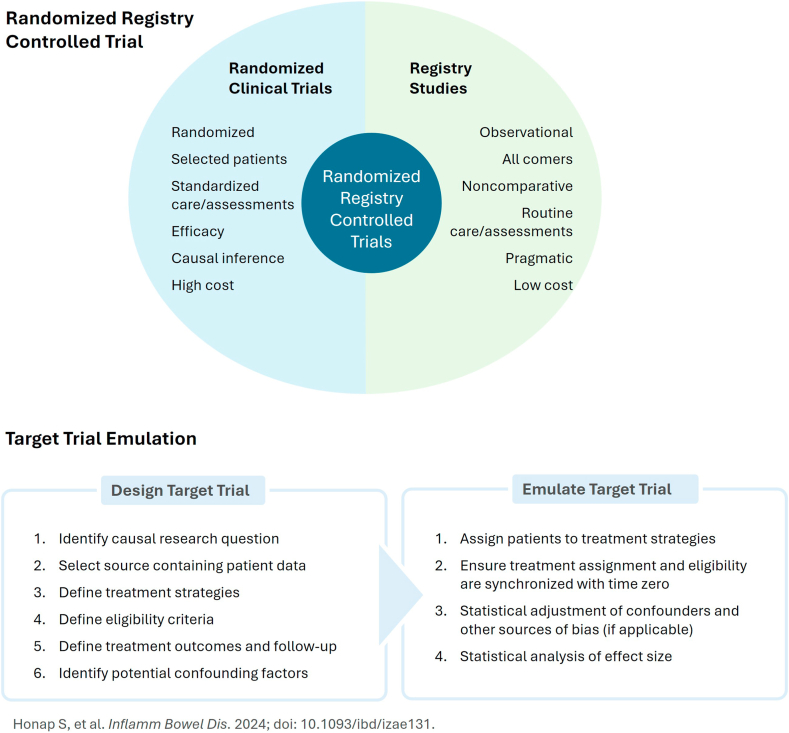
**Pragmatic trial designs**.^17^

Large data sets collected through electronic health records and administrative health databases offer an important complement to prospective registry studies. These “lean” options can be utilized to generate cost-efficient, timely, high-quality evidence without conducting more costly RCT or prospective registry studies (Figure 1).^17^ Although often performed as observational studies, there are options to apply unique methodologies to generate comparative data, such as the randomized registry controlled trial^18^ (eg, TASTE,^19^ SWEDEPAD,^20^ VSTAR-1^21^). These registry-based trials use data, including patient characteristics and outcomes, that are already being collected and can embed randomization into different treatment strategies to determine comparative outcomes. In this model, study visits are aligned with routine clinical visits, study-specific data are collected via the registry interface, and patients can be recruited faster at fewer sites and at a lower per-patient cost. The lower visit burden and absence of duplicative data collection mean less inconvenience for patients, physicians and site staff alike. Another option is the target trial emulation methodology, which applies the principles of an ideal, or “target,” randomized trial to data routinely collected in clinical practice.^17^^,^^22^ This approach starts with a well-defined research question, considers a theoretical randomized trial (the “target trial”), establishes a study protocol (ie, eligibility criteria, end point definitions, etc), and emulates randomization of patients to control for confounding.

### Primary end points for CLTI device trials: Which ones should we be using?

Much debate exists around the optimal primary end point(s) for trials evaluating novel devices to treat CLTI. The rigorous RCT of drug-coated or eluting devices in PAD (IMPERIAL, IN.PACT SFA, LEVANT-2, ILLUMENATE, and LIFE-BTK) had 12-month primary patency as the primary efficacy end point (LIFE-BTK actually had a composite of limb salvage and primary patency). Debate continues as to whether primary end points should be clinical (eg, clinically driven target lesion reintervention [CD-TLR], wound healing, major amputation, acute limb ischemia), image-based (eg, binary restenosis, patency), patient-centric (related to patient symptoms eg, patient-reported outcome measures [PROMS]), or composite outcomes (Table 2). While symptom improvement is the primary objective of treatment, patient-reported end points, such as QOL and PROMS, can be challenging to interpret and quantify. Furthermore, no specific PROM or QOL measure has been designed for the CLTI patient. As a result, these critically important end points often become secondary or exploratory trial end points.

**Table 2:** Examples of end point considerations in PAD trials.

End points	Pros	Cons	Options
QOL/PRO end points			
Improvement in QOL/PRO scores (eg, EQ-5D-5L)	• Ultimate goal • Used as secondary end points to show improvement from baseline	• Least specific • Lack specific tools designed for CLTI patients	• Include in a composite primary end point • Invest in the development of a CLTI-focused PROM • Continue to use as secondary end points
Clinical end points			
Major amputation	• Drives treatment decisions • More specific to limb outcomes	• Low frequency event rate • Challenges in adjudicating whether amputation is due to vascular or nonvascular conditions (eg, persisting infection)	• Composite primary end points to enrich frequency of total events • Hierarchical composite end points and win ratios to prioritize outcomes more critical to CLTI care • Longer primary end point assessment (eg, 12 months instead of 6 months) • Larger sample sizes to increase event rates • Include more severe PAD patients (eg, Rutherford class 5/6) to increase event rates • Independent adjudication and oversight to improve standardization is important
TLR/TVR			
Symptom relief (eg, pain improvement)	• Patient-focused	• Subjective changes in pain can be hard to capture and are transient	
Wound healing	• Patient-focused	• Nonstandardized wound assessment and care • Wounds may fail to heal for reasons unrelated to adequate blood flow (eg, infection)	
Amputation-free survival	• Ultimate end point capturing both major morbidity and mortality	• Death may be due to non–CLTI-related causes • Obfuscates granularity as to limb status and function	
Imaging end points			
Patency /binary restenosis	• Readily available • Standardized and reproducible • Support mechanism of the action of device • Increase end point rates	• Not always concordant with clinical outcomes • Challenging to isolate the impact of individual device • Challenging to incorporate adjunctive therapies (eg, atherectomy) • Performance can suffer from human variability	• Prioritize hierarchical composite outcomes • Independent adjudication and oversight to improve standardization is important

CLTI, chronic limb-threatening ischemia; PAD, peripheral artery disease; PRO, patient-reported outcome; PROM, patient-reported outcome measure; QOL, quality of life; TLR, target lesion reintervention; TVR, target vessel reintervention.

Clinical end points, such as major amputation, acute limb ischemia, freedom from CD-TLR, symptom relief, and wound healing, are specific and patient-focused outcomes, but have limitations. For instance, CD-TLR may not always be driven by patient symptoms and can lack objectivity in single-blinded trials, where the treating physician may know the device assignment. In addition, CD-TLR may not capture lesion subsets that are procedurally challenging to treat and are instead managed medically, such as an occluded vein graft in a patient with stable symptoms. Freedom from major amputation is a critical end point but is less practical as a primary outcome because of its low frequency, which would result in requiring a large sample size to power an RCT. In addition, it can be challenging to adjudicate whether the need to amputate is related to PAD or other competing conditions (eg, persistent infection). Wound healing is also very important to CLTI patients and treating clinicians, but wound care and assessment are not standardized in clinical practice; thus, it is difficult to standardize in trials unless done under the oversight of a wound core lab. Furthermore, poor wound healing can be a result of many factors, including tobacco use, diabetes control, nutrition, and infection, and thus may not directly represent an improvement in perfusion related to the tested device.

Imaging end points can be standardized by core laboratories and can confirm a treatment’s mechanism of action (eg, restoration of flow or patency). However, discordance between patency and clinical improvement is common, as many patients may lack symptoms even with loss of patency. Furthermore, human variability in acquisition, in particular for Doppler ultrasound (DUS), can create quality challenges. Among imaging end points, binary restenosis has recently become the preferred outcome for BTK intervention trials, similar to what has been used for above-the-knee interventions.^23^ In the tibial vessels, DUS can be utilized when stents and scaffolds are present and has shown good agreement with angiography above the knee and moderate agreement with BTK in blinded analyses.^24^ Further confirmatory validation studies of appropriate thresholds to identify the severity of stenosis (ie, peak systolic velocity ratio >2.0 vs >2.4) are needed.

Composite outcomes and coprimary end points are 2 widely used alternatives to selecting a singular primary end point. Coprimary end points allow for safety and efficacy to both serve as primary end points with equal standing and account for competing risks (eg, a patient dies prior to undergoing amputation for a nonviable limb). However, these often require more power for each end point, and thus, larger sample sizes. Traditional composite end points (eg, major adverse limb events, major adverse cardiac events) have several limitations: multiple components may dilute the interpretation of the result; equally weighted components even though not all events are equal (eg, nonfatal events ≠ fatal events); 1 high frequency end point (eg, binary restenosis) can drive the outcome; and definitions of a composite end point that are not standardized across trials, such as major adverse limb events.^25^^,^^26^ In addition, censoring after the first event results in greater importance of more frequently observed and nonfatal events over events that may be more meaningful (eg, loss of patency occurring earlier vs subsequent death later in hospital course). The ACHILLES and LIFE-BTK trials have demonstrated the value of composite end points for capturing comparative efficacy in PAD trials. These include primary patency (defined as the absence of CD-TLR and binary restenosis) in ACHILLES and the 4-part composite end point in the LIFE-BTK trial (freedom from target limb above-the-ankle amputation, total occlusion of the target vessel, CD-TLR, and binary restenosis of the target lesion).^27^^,^^28^ Both trials also demonstrated the importance of evaluating efficacy end points out to 12 months rather than 6 months.

**Figure 2:**
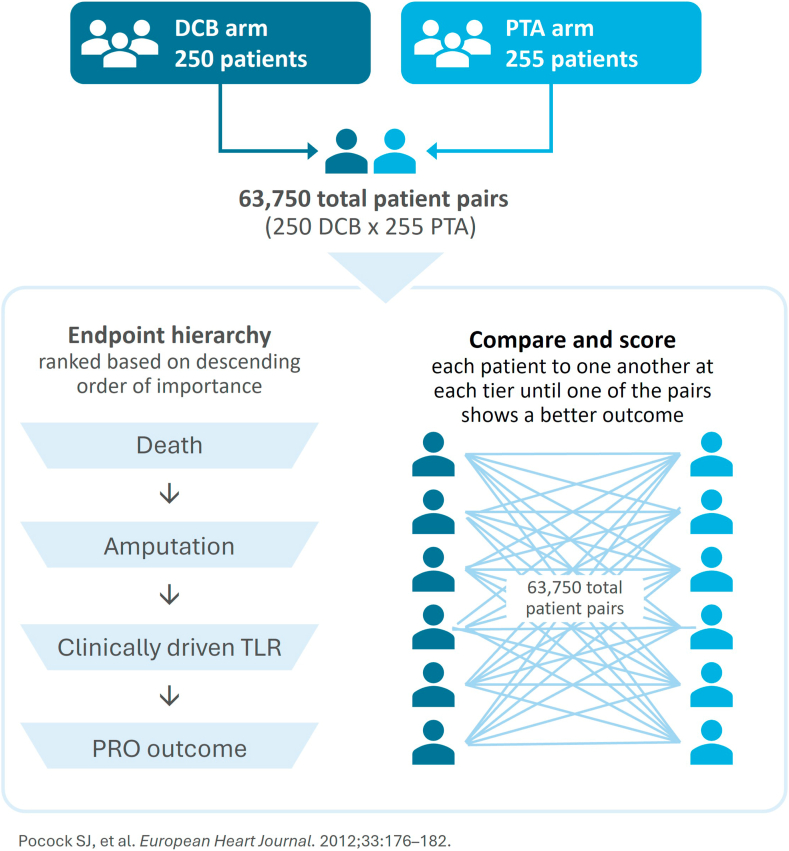
**Multiple pairwise comparisons in a win ratio analysis.****^29^** Each patient in the percutaneous transluminal angioplasty (PTA) arm is compared to each patient in the drug-coated balloon (DCB) arm. PRO, patient-reported outcome; TLR, target lesion reintervention.

Win ratio analysis is an emerging statistical methodology that may offer a solution to adding weight to endpoints that are more clinically significant. The win ratio creates a hierarchical composite end point that can account for the relevance and importance of the component end points, as well as multiple events and time to events. This approach, which involves multiple pairwise comparisons (Figure 2), increases the power to detect a treatment difference in a relatively small sample by using all available events.^29^ Composite imaging-clinical end points (eg, primary patency + freedom from amputation) could also be analyzed with this approach.

Regardless of the chosen end points for CLTI trials, the use of adjunctive devices in trials remains a challenge. On the one hand, the goal of evaluating a new device is to measure how it performs in clinical practice; restricting devices that are commonly used in conjunction with the trial device (eg, atherectomy prior to drug-coated balloon) can limit generalizability. Conversely, it may be difficult to isolate the impact of a device from adjunctive therapies and known or unknown confounders, such as wound care, lesion characteristics (eg, occlusion, length, calcification, inflow disease), comorbidities (eg, glycemic control, uremic control), and demographic factors (eg, age, sex).

## Patient selection: Who should be enrolled in a CLTI device RCT?

Eligibility criteria are important in any study. Excluding more severe or complex patients is an established approach to simplifying analysis and isolating the impact of the treatment under investigation. This is particularly relevant in CLTI trials when considering enrollment of patients with high probabilities of major amputation, as this risk factor would obfuscate the assessment of study device performance. However, restricting trial populations through tight inclusion criteria removes what many consider “real-world” patients, including those with severe disease and complex comorbidities, and often results in high ratios of screened to randomized patients. This approach slows enrollment, reduces sample sizes, lowers event rates, and creates low-complexity, homogeneous trial populations that do not reflect many patients encountered in daily practice. Modifying patient selection criteria has been done in some clinical trials to capture higher-risk patients without incurring the risks of a highly probable end point; for example, a trial may select Rutherford class 6 patients with specific parameters (eg, without persistent osteomyelitis or high-risk locations of tissue loss requiring a greater likelihood of major amputation). Additionally, restricting the proportion of Rutherford class 4 patients allowed in a trial is another strategy to ensure a broad distribution of CLTI disease severity. Other challenges with strict inclusion criteria are the resulting loss of ethnic, racial, and gender diversity. Minority patients in particular often have more frequent exclusionary comorbidities (ie, end-stage renal disease on dialysis) or have language barriers that prohibit enrollment. Significant efforts by regulators and sponsors have worked to address these challenges in recent years, as highlighted by the LIFE-BTK trial.^30^

## BTK: What is so different?

Additional considerations for designing head-to-head studies in the BTK setting include what role commercially available adjunctive devices should have, including scaffolds, vessel prep tools, and intraprocedural and postprocedural imaging. Prior to approval of the Esprit resorbable drug scaffold in April 2024, there were no FDA-approved stents for BTK, though coronary stents have been used in these vessels over the past decade in off-label fashion. Controlling for the use of nonindicated devices is challenging; considerations include strict anatomic and clinical criteria, usage consistent with the standard of care and routine practice patterns, and distinguishing between device success and treatment success (eg, stent-free patency).

Despite increasing use of atherectomy as an adjunct to percutaneous transluminal angioplasty (PTA), comparative outcomes with vs without adjunctive atherectomy in BTK interventions are limited, as these devices have been excluded from most contemporary trials.^11^ Vessel prep with atherectomy may offer both proven and theoretical benefits to improving procedure outcomes, including the ability to reduce elastic recoil, increase lumen size, modify calcified plaque, improve uptake of antiproliferative drugs, prevent dissection, reduce stent fracture, and optimize stent apposition and expansion.^31^ The Excellence in Peripheral Artery Disease (XLPAD) Registry suggests that adjunctive atherectomy in BTK interventions is associated with lower rates of 1-year repeat target limb revascularization.^32^ Atherectomy is commonly used during BTK revascularization in the US, and its exclusion can hamper the generalizability of tested devices, such as atherectomy use with a drug-coated balloon. However, the combination of devices can complicate the interpretation of findings, which creates challenges for regulatory authorities.

Retrograde access is often used during BTK revascularization and allows endovascular treatment of occlusions that span the femoropopliteal segment through the trifurcation of the tibial vessels.^33^ However, the need for a retrograde approach often reflects increased procedure complexity and disease severity. The disadvantages of this approach are that it can compromise tibiopedal arteries, in particular when single vessel run-off is present, and it does not allow for adequate imaging at the completion of the procedure if single access is used. A recent Cochrane systematic review was unable to identify any randomized or quasi-RCTs comparing retrograde distal access vs femoral access for BTK intervention.^34^ The LIFE-BTK trial’s inclusion of retrograde access for lesion crossing or re-entry accommodated the general trend in use in clinical practice and facilitated improved enrollment, but it limited the ability to treat from the retrograde access, thus also requiring femoral access. This facilitated the use of lower-profile sheaths or sheathless techniques and provided completion imaging following intervention.^30^

There are several considerations for imaging techniques in the BTK setting. Although the use of intraprocedural intravascular ultrasound (IVUS) imaging in peripheral interventions is just emerging, increasing evidence suggests that these imaging modalities may improve outcomes of peripheral endovascular interventions.^35^, ^36^, ^37^ Most importantly, IVUS can allow for more precise vessel sizing, which is critical for use of drug-coated technology and scaffold implants. In a clinical trial setting, IVUS can assess stenosis better than angiography, even when quantitative vascular analysis is used.^38^ One of the challenges of IVUS in clinical trials is standardizing operator expertise and technique, as well as comparison to postprocedure angiography. For example, the LIFE-BTK trial showed more accurate vessel sizing, balloon size, and greater acute gain in IVUS-guided vs quantitative angiography–guided endovascular interventions.^38^ LIFE-BTK’s protocol strongly encouraged the use of IVUS, developed an implant technique that optimized methods for sizing and wall apposition, and collected data on the IVUS subset.

Patency is necessary for demonstrating the superiority of one intravascular device or technique over another. In BTK vessels, noninvasive DUS is preferred over angiography for patency assessment, except in the presence of severe diffuse calcification, a common infrapopliteal disease pattern associated with diabetes and end-stage renal disease. DUS and angiography have important complementary roles in evaluating patency in BTK intervention trials. DUS is physiologically relevant, noninvasive, painless, inexpensive, readily available, and rapid, whereas angiography is objective but invasive, timely, costly, and comes with risks to the patient. Transitioning to DUS for the assessment of patency and restenosis over angiography alone is an important change that has helped improve enrollment in BTK studies in the US, but such a transition requires continued validation of DUS's efficacy vs angiography and standardized approaches for core imaging laboratories.

## Optimal medical therapy: Why device trials need it?

Indpendent of endovascular or surgical revascularization, the known drivers of PAD progression (diet, smoking, sedentary lifestyle, inflammation, diabetes, obesity, and genetics) must be addressed for treatment to succeed in the long term. There is strong evidence supporting the role of smoking cessation, dyslipidemic therapy, antiplatelet therapy, anticoagulants, and antihypertensive agents in improving long-term limb outcomes.^39^^,^^40^ Despite evidence that statin and antiplatelet therapy are effective in both early and advanced PAD,^41^ both are underutilized and not well adhered to among PAD patients relative to coronary artery disease patients (who also fare better).^42^, ^43^, ^44^, ^45^ Only 14% of PAD patients who undergo endovascular or surgical procedures are discharged on all 4 guideline-directed therapies,^44^ despite the fact that rates of major adverse limb and cardiac events are lower when all guideline-directed therapies are utilized.^46^ The proportion of patients discharged on guideline-directed therapy could be an informative clinical end point in PAD intervention trials, and it should be the responsibility of trial leadership to improve medical adherence among this vulnerable patient population.

## Coming to consensus on CLTI device trial design

The treatment of CLTI, particularly the achievement of limb salvage, remains challenging regardless of approach. Multispecialty care is essential, given that endovascular and surgical interventions only address a fraction of the factors that affect disease pathogenesis and progression. Progress in treating CLTI is iterative and will require evidence from both RCT and real-world evidence studies. It will be important for CLTI intervention studies to measure factors of critical importance to patients (ie, amputation, wound healing, and death), and mechanistically determine how interventions improve limb-threatening ischemia (ie, patency). The focus on the patient should not be forgotten, and patient-reported outcomes will continue to be critical secondary end points to evaluate device performance.

In summary, this Vascular Leaders Forum on CLTI care identified 4 broad strategies to optimize comparative trials evaluating CLTI interventions (Central Illustration). First, primary end points should be chosen with attention to clinical, patient-centric, imaging, and hierarchical design considerations. Ideally, end points should be standardized in a manner similar to the Vascular InterVentional Advances performance goals for SFA^47^ and should include prescription of guideline-directed medical therapy. Regulatory agencies could provide guidance here based on their combined experience with end point selection across many trials. Second, broader eligibility criteria can expand and hasten enrollment and are important for gathering evidence on outcomes in the patients often encountered in real-world practice, in particular underrepresented minority populations that are often more medically complex. Third, there continues to be a movement for extending the primary end point timing to 12 months with additional follow-up out to 24 to 60 months, allowing for a longer period of device evaluation and the ability to enrich clinical end point rates. Finally, innovative pragmatic trial designs and statistical methodologies are needed to conduct comprehensive, cost effective, relevant trials with sufficient statistical power but without prohibitively large sample sizes and study durations. Overall, the goal of future device trials for revascularization of CLTI is to better capture a broad PAD population and a focus on patient-centric outcomes, maintaining alignment with global vascular guidelines,^1^ while also balancing both pragmatism and costs. Success will require all stakeholders, including regulators, multidisciplinary clinicians, patients, and sponsors.

**Central Illustration:**
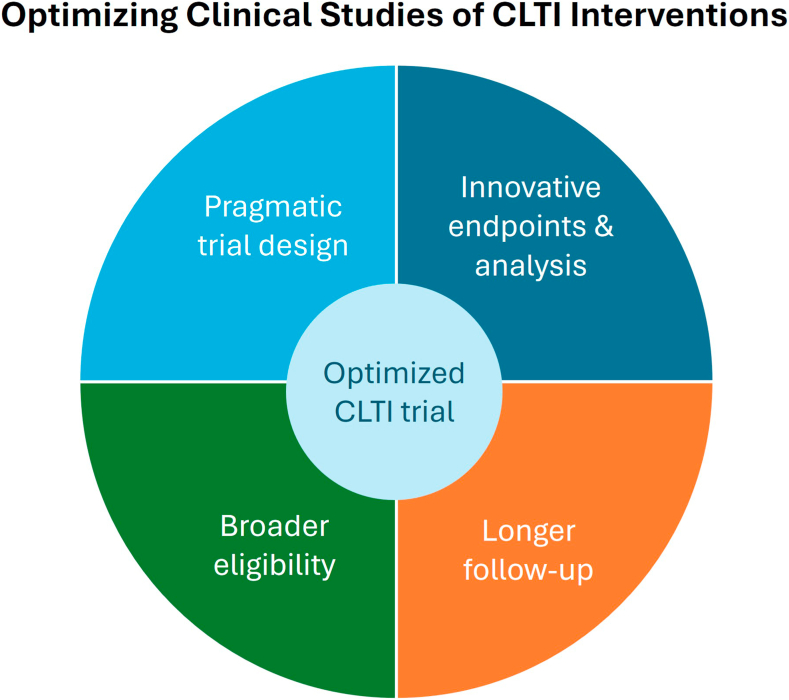
Optimizing clinical studies of chronic limb-threatening ischemia (CLTI) interventions.
