# Supplementary material for: Contemporary Chronic Limb-Threatening Ischemia Care in the United States—Part 2: Designing Clinical Device Trials
Source: J Soc Cardiovasc Angiogr Interv. 2025 Nov 3;4(12):103934. doi: 10.1016/j.jscai.2025.103934 (PMC12766039; doi:10.1016/j.jscai.2025.103934)
Supplement: Supplementary Material [file mmc1.pdf]

VIVA Foundation 2024 Vascular Leaders Forum

List of Participants

George Adams, MD, MHS, MBA
David Armstrong, DPM, PhD
Ehrin Armstrong, MD
Swamy Avasarala
Seshagiri Rao Avasarala
Danielle Bajakian, MD
Joshua Beckman, MD
Donna Buckley, MD
Venita Chandra, MD
Daniel Clair, MD
Tony Das, MD
Brian DeRubertis, MD
Bryan Fisher, MD
Eri Fukaya, MD, PhD
Elizabeth Genovese, MD
Patrick Geraghty, MD
William A. Gray, MD
Caitlin W. Hicks, MD, MS
Michael R. Jaff, DO
John A. Kaufman, MD
Raghu Kolluri, MD, MS
John R. Laird, MD
Alexandra Lansky, MD
Robert Lookstein, MD, MHCDL
Steven Lossef, MD
Sean Lyden, MD
Kumar Madassery, MD
Misti Malone, PhD
Jay Mathews, MD, MS
Kym McNicholas
J.D. Meler, MD
Matthew Menard, MD
D. Christopher Metzger, MD
Zola N'Dandu, MD
Leigh Ann O'Banion, MD
Parag J. Patel, MD, MS
Constantino Peña, MD
Brian Pullin
Mahmood Razavi, MD
Kenneth Rosenfield, MD, MHCDS
Vincent Rowe, MD
John H. Rundback, MD
Saher Sabri, MD
Eric A. Secemsky, MD, MSc
Mehdi Shishehbor, DO, MPH, PhD
Niten Singh, MD
Peter A. Soukas, MD
Megan Tracci, MD
Julie Tyler
Bret N. Wiechmann, MD
Bram Zuckerman, MD
